# Perceptions of Emergency Care by Sexual and Gender Minorities in Colorado: Barriers, Quality, and Factors Affecting Identity Disclosure

**DOI:** 10.5811/westjem.2021.3.49423

**Published:** 2021-07-14

**Authors:** William G. LaPlant, Leonardo Kattari, Lexie K. Ross, Jennifer Zhan, Jeffrey P. Druck

**Affiliations:** * Good Samaritan Medical Center, Department of Emergency Medicine, Brockton, Massachusetts; † Michigan State University School of Social Work, East Lansing, Michigan; ‡ University of Colorado School of Medicine, Aurora, Colorado; § California Hospital Medical Center, Department of Emergency Medicine, Los Angeles, California; ¶ Public Health Institute California Bridge Program, Oakland, California; || University of Colorado School of Medicine, Department of Emergency Medicine, Aurora, Colorado

## Abstract

**Introduction:**

Expanding on data concerning emergency department (ED) use and avoidance by the sexual minority (those who identify as lesbian, gay, bisexual, queer, other [LGTBQ+]) and gender minority (those who identify as transgender, gender nonconforming, other) community may inform future ED LGTBQ+ training and clinical practice. Investigation objectives included characterizing rates of emergency care avoidance, identifying barriers to emergency care, and assessing emergency care quality and cultural competency for sexual and gender minorities.

**Methods:**

In this population-based, cross-sectional needs assessment, sexual minority, gender minority, and/or cisgender heterosexual-identified participants were selected based on participants’ subscription to newsletters or social media accounts for One Colorado, an LGBTQ+ advocacy organization. Each participant completed a single digital survey that collected qualitative and quantitative data about ED perception, use, and demographics.

**Results:**

A total of 477 LGBTQ+ or heterosexual-identified individuals (mean age = 44.3 (standard deviation [SD] = 16.7)) participated in the study. Lifetime emergency care avoidance rates for gender minorities were markedly increased (odds ratio [OR] 3.8, 95% confidence interval [CI], 2.2 – 6.6; P <.001), while avoidance rates for sexual minorities were similar to those of cisgender heterosexual respondents (17% vs 14%; P <.001). Gender minorities were more likely than sexual minorities to both avoid emergency care due to fear of discrimination (43% vs 15%; P =.002) and to have experienced discrimination during their last ED visit (OR 11, [95% CI, 5–24]; P <.001). No significant differences were observed between participants in care avoidance due to financial reasons or prior negative experiences. No cited ED factors that influenced identity disclosure decisions were distinctly predictive.

**Conclusion:**

Gender minorities are more likely than sexual minorities and heterosexual cisgender individuals to report ED avoidance and discrimination at last ED visit. Future work characterizing deficits in LGBTQ+ ED care might reduce these avoidance and discrimination rates, enhancing the level of patient care provided to this population.

## INTRODUCTION

Despite significant advances in lesbian, gay, bisexual, transgender, queer, and other sexual and gender minority-identified (LGBTQ+) rights, sexual minorities (those who identify as lesbian, gay, bisexual, queer, other) and gender minorities (those who identify as transgender, gender nonconforming, other) still experience considerable socioeconomic disparities that impact health and healthcare access. Fear of discrimination often frames this population’s healthcare encounters and shapes healthcare-related behaviors.^1^,^2^

Limited published data characterizes the LGBTQ+ population’s access to emergency medical care. A focused study of sexual minorities in the Bronx found that emergency department (ED) use was higher compared to the general population, despite adequate access to primary care physicians. Nearly 78% of LGB individuals surveyed had a primary care doctor with whom they were comfortable discussing LGB issues.^3^ Conversely, a Canadian study found 21% of transgender respondents reported previously avoiding emergency care due to fear that their identity would affect their care.^4^ A subsequent study supported the Canadian investigation, finding gender minority-identified participants more likely to report negative effects of identity disclosure to their provider.^5^ Despite these foundational investigations, no prior study has provided detailed data on care avoidance for both sexual and gender minorities, care satisfaction, and factors associated with identity disclosure in the emergency care setting.

One investigation exploring gender minority-identified individuals’ ED experiences found an association between negative ED experiences and lack of provider sensitivity toward and training about this population.^6^ When exploring cultural competency training in the ED, a survey of emergency medicine residency program directors found that only a third incorporated LGBTQ+ health content into the didactic curriculum.^7^ An additional survey of physicians at an academic health center found that the majority of physicians would not regularly discuss sexual orientation, sexual attraction, or gender identity with patients.^8^ Taken together, the paucity of culturally competent LGBTQ+ training for residents and limited incorporation of LGBTQ+-relevant discussions into patient care pose potential barriers to providing optimal care for LGBTQ+-identified individuals. The need for culturally competent ED care is critical given the significant use by minority patients and the rapidity of the work, where brief contact time and the need for efficiency can magnify small discordances in patient interactions.^9^ To adequately address the needs of minority patients within the ED, we must first develop a robust understanding of those needs.

With this needs assessment, we sought to identify the following: care avoidance rates and factors associated with care avoidance; factors associated with ED selection for the LGBTQ+ community; factors associated with identity disclosure within the ED; and factors associated with perceived discrimination in the ED.

**Population Health Research Capsule**

What do we already know about this issue?

*Members of the sexual and gender minority (SGM) community experience considerable socioeconomic disparities that impact their health and healthcare access*.

What was the research question?

*How do SGM community members perceive emergency department (ED) care relative to heterosexual individuals?*

What was the major finding of the study?

*Gender minority community members reported ED avoidance and discrimination more than other study participants*.

How does this improve population health?

*Efforts to reduce rates of ED avoidance and perceived discrimination among SGM community members could enhance the level of care provided to this population*.

## METHODS

### Study Design and Population

Surveys were distributed through the email list and social media accounts of a prominent Colorado LGBTQ+ organization, One Colorado, from August–November 2015 using three separate email notifications. Anyone with a survey link was eligible to participate, although Colorado residents belonging to the LGBTQ+ community were specifically targeted through the selected distribution method. The study was determined to be exempt from review by the University of Colorado Institutional Review Board.

### Survey Content and Administration

A digital survey used 36 multiple-choice questions and fill-ins to collect qualitative and quantitative data about ED perception and use, as well as demographic information. We used a validated, two-question approach to assess gender and assign participants to the gender minority group.^10^ The remainder of the questions were designed by the study team, tested within the survey group, tested on two external volunteers, and revised extensively over a one-month period based on feedback about clarity of questions and concern regarding answer options.

### Data Analysis

Data were housed in a Microsoft Excel document (Microsoft Corporation, Redmond, WA) and analyzed using Stata 13 (StataCorp, College Station, TX), using chi-squared and Fisher’s exact tests (when n <10 in a given cell) to assess for differences in categorical data according to a predetermined statistical analysis plan. Participants were excluded if both sexual orientation and gender identity were not reported. Based on key results from this analysis, alongside pre-hoc hypotheses, we used logistic regression to determine odds ratios (OR) for care avoidance and reporting a negative last ED visit. Logistic models were built additively based on *P*-value and effect size from a model containing all factors hypothesized to generate an effect.

## RESULTS

The survey was distributed to a listserv of 10,000 members of a local LGBTQ+ organization, with requests to respond about their personal experience in the emergency department. A total of 477 participants who reported gender and sexual orientation responded to the survey; however, as the total number of members who fit those criteria is not known, an actual response rate cannot be calculated. Of these participants, 450 completed meaningful portions of the survey. The final sample consisted of six heterosexual men, 36 heterosexual women, 168 sexual minority men, 150 sexual minority women, and 90 gender minorities (22 transgender men, 34 transgender women, and 34 gender nonconforming, intersex, or other respondents). Of those responding, 88% had previously visited the ED, with an average time since last ED visit of 5.3 years (standard deviation = 6.7). Further summary statistics are available in Table 1.

Gender minorities reported higher rates of ED avoidance compared to sexual minorities and heterosexual cisgender respondents. Sexual minority respondents reported similar rates of ED avoidance compared to their heterosexual peers; however, the sampling technique combined with low numbers of heterosexual respondents may limit the conclusions that can be drawn about heterosexual cisgender groups. There was no difference in avoidance rates between heterosexual and sexual minority women (*P* = 0.382). Small numbers of male heterosexual cisgender respondents limited the ability to assess for differences between heterosexual and sexual minority men.

Care avoidance was additionally associated with both annual income level (*P* <0.001) and insurance type (*P* = 0.003). No difference in avoidance rates were noted between White and non-White respondents (*P* = 0.115). Gender minorities were more likely to have a lower income than sexual minorities (*P* <0.001) and less likely to have private insurance (*P* <0.001). Using logistic regression to control for the effect of income, insurance type, and race revealed that the odds of reporting care avoidance were 3.8 times greater among gender minorities compared to male sexual minorities (Table 2).

Half of those who previously avoided care reported doing so for financial reasons, with an equal distribution for heterosexual cisgender respondents, sexual minorities, and gender minorities (Table 3). Fear of discrimination was identified as a barrier to seeking care more frequently by gender minorities (43%, 19/25) compared to sexual minorities (15%, 8/55) (*P* = 0.002). Of those with a history of care avoidance, 45% (20/44) of gender minorities and 29% (16/55) of sexual minorities reported a prior negative ED experience as the reason for avoidance (*P* = 0.241). The rate of avoidance due to a prior negative experience outside of the ED was again similar between sexual and gender minorities (*P* = 0.248).

When choosing an ED, few respondents reported researching the ED to determine its LGBTQ+-friendliness prior to presenting (3%, 10/356). Gender minorities were more likely to research departments (7%, 6/83) than sexual minorities (1.5%, 4/273) (*P* = 0.028). Factors affecting ED choice included proximity (53%), transport by emergency medical services or another individual (20%), reputation (9%), and other reasons such as insurance limitations (15%). Only 1% of respondents chose an ED based on the knowledge of its LGBTQ+-friendliness (one gender minority, four male sexual minorities).

Of those who felt the question was applicable, 36 respondents (12%) felt their LGBTQ+ identity negatively affected their most recent visit, with 41% of these respondents believing they were treated differently than other patients and 41% reporting hearing homophobic/transphobic language in the ED. Respondents who self-identified as LGBTQ+ parents also reported difficulties presenting with a child for care, including needing to correct staff on correct pronoun usage.

*“I’ve been expected to coach attending medical staff on pronouns and grammar while receiving emergency care and also while being interrogated (and argued with) about my biological sex (because they didn’t understand being intersex nor did they understand the difference between gender and sex).”*

Some cisgender sexual minority respondents noted subtle ways in which they felt marginalized by staff.

*“My husband and I went together to the ER. No one attempted to keep us apart but no one inquired as to our relationship either. In some ways this ‘avoidance’ of our relationship made me feel a bit awkward. When physicians and nurses spoke to me, they basically ignored my spouse. It would have been better if they asked what our relationship was and then indicated approval and spoke to both of us as a couple. It has been my impression that with straight couples they tend to speak to both husband and wife as a pair.”*

*“I was there of [sic] a relatively minor emergency room procedure, and had my girlfriend accompanying me while I was there. After her being repeatedly referred to as my friend, we both felt more comfortable if she wasn’t sitting directly next to me when the doctor came in.”*

Gender minorities were 10 times more likely than sexual minorities to report their identity negatively affecting their last visit (35% vs 5%) (95% CI, 5–24, *P* <0.001). Reporting a negative visit was more prevalent in the lowest third of income (<$35,000, 21%) vs the middle- (6.7%) and high- (8.1%) income groups (*P* = 0.005). Similar income- and access-related trends in identity negatively affecting a respondent’s last visit were observed in those with Medicaid (30%) compared to those with Medicare (13%) or private (8.2%) insurance (*P* = 0.008).

No significant differences in rates of reporting a sexual or gender identity-associated negative ED experience were noted based on race (11% of White respondents and 19% of non-White respondents, *P* = 0.168) or in sexual minorities based on gender (3% of men and 7% of women, *P* = 0.234). No significant differences in reporting a negative last ED visit were observed for those who presented for a mental health (24% vs 11%, *P* = 0.122) or sexual health (0 vs. 12%, *P* = 1) concern.

Respondents cited numerous intra-ED factors that impacted comfort with their sexual and/or gender identity disclosure. Excluding subjects who selected both positive and negative options (n = 13), the following were selected by respondents as positively or negatively impacting their decision to disclose identity in the ED: welcoming (22%) or unwelcoming (9%) nurse; welcoming (18%) or unwelcoming (7%) physician; non-inclusive intake forms (ie, binary gender options) (21%); lack of LGBTQ+ signage (20%); and lack of gender- neutral bathrooms (10%). Respondents cited non-inclusive non-discrimination statements, presence of family members to whom the patient hadn’t disclosed their identity, and negative experiences with administrative staff as additional factors affecting identity disclosure.

Analyzed as three levels (factor not commented on, supportive factor noted, and detracting factor noted), the presence of a nurse or physician non-comment, supportive comment, or detracting comment was associated with increased likelihood of identity disclosure (Table 4).

While positive and negative ED factors were minimally predictive of identity disclosure, they were markedly predictive of whether or not a respondent’s sexual/gender identity negatively impacted the last ED visit. Assessed independently, there were differences (*P* <0.001) in reporting a negative experience depending on having a welcoming/unwelcoming physician or nurse, presence of gender-neutral bathrooms, absence of an inclusive intake form, or absence of LGBTQ+ signage. Assessed together using logistic regression and controlling for whether or not the patient was a gender minority, odds of reporting a negative visit were increased by having an unwelcoming physician (OR [4.4], *P* = 0.035) or nurse (OR [22], *P* <0.001), with no effect based on the presence or absence of LGBTQ+ signage, gender-neutral bathrooms, or inclusive intake forms (Table 5). Neither income level nor insurance type contributed to this model, and so were excluded.

## DISCUSSION

This needs assessment uncovered a variety of data on ED utilization by the LGBTQ+ community and revealed several important findings.

### Emergency Department Avoidance

In both sexual minorities and gender minorities ED avoidance was prevalent when controlling for income, insurance, and race, gender minorities avoided more frequently, consistent with a previous investigation conducted in the United States.^6^ Among those who reported a history of avoidance, a similar proportion of sexual and gender minorities avoided for financial reasons or due to prior negative healthcare experiences. Gender minorities reported avoiding due to fear of discrimination more than sexual minorities. Expressed as a proportion of all gender minorities, the percentage who reported avoidance due to fear of discrimination was identical to the previously reported percentage of trans community members who avoided care in Ontario, Canada.^4^ However, limited conclusions about ED avoidance can be drawn between these two studies due to differences in sampling methodology.

Our findings suggest that emergency care avoidance within the gender minority community occurs significantly more often than in the heterosexual cisgender community. Prior negative ED experiences as well as perceptions about ED care beyond personal experience appear to shape this behavior. These findings suggest a need to couple improving intra-ED care with outreach and public efforts, such as involving local LGBTQ+ organizations in physician training.

### Emergency Department Choice

While few respondents researched the ED to determine its LGBTQ+-friendliness prior to presenting, gender minorities reported this behavior more frequently than sexual minorities. Respondents reported having little control over ED choice, with the majority choosing based on proximity, extrinsic factors (eg, insurance coverage), or transport by emergency medical services. A portion of respondents chose based on reputation, which may have incorporated LGBTQ+-friendliness and thus diluted the response for choosing an ED specifically for this quality.

These findings suggest that services for identifying LGBTQ+-friendly providers or hospitals, while useful in primary care and for elective procedures, may be less beneficial in emergency care. If online resources for identifying LGBTQ+-friendly EDs were to be developed, our results suggest that emphasis should be placed on EDs committed to quality, gender minority care, such as those requiring cultural competency staff training.

### Identity Disclosure

Respondents reported many ED factors that contributed to identity disclosure. A welcoming physician or nurse was the most common hospital factor that contributed to disclosure, while lack of LGBTQ+ signage or non-inclusive intake forms (eg, binary gender options) were detracted from identity disclosure.

When analyzed as a three-level variable (factor not commented on, positive factor noted, detracting factor noted), noting any factor was associated with identity disclosure at last visit regardless of emotional valence. This might suggest recall bias, where those with a positive or negative experience during their last ED visit were more likely to recall factors that those with a neutral experience, thus affecting our ability to measure a true relationship. This may also represent an element of reverse causation; those who disclosed their identity might have been more likely to experience negative encounters with staff. Given these analytic complications, the true impact of these factors on identity disclosure is difficult to assess.

Additional factors that were not captured by our survey likely impact identity disclosure decisions. While not predictive of identity disclosure as a binary yes/no, our data do suggest that patients analyze numerous intra-ED factors as part of the identity-disclosure process.

### Emergency Department Discrimination

Gender minorities were more likely than sexual minorities to report a last visit negatively impacted by gender identity/sexual orientation. Negatively perceived interactions with physicians and/or nurses were strongly predictive of experiencing a negative encounter. Increased estimates for a negative interaction with nurses vs physicians may reflect longer interaction time with nurses, interactions with multiple nurses, or more overt differences in care quality based on discrimination. These results may also reflect chance, with similar odds of causing a negative experience when accounting for the wide confidence intervals.

Our findings suggest that interventions aiming to improve LGBTQ+ cultural competency should target both physicians and nurses. No information was gathered regarding negative encounters with other ED staff (eg, administrative staff, technicians, etc.); so data-based recommendations cannot be made for this group.

No differences were observed in the likelihood of discrimination based on race, gender among sexual minorities, or visit type, although the relatively low event rate and number of subgroups analyzed limits definitive conclusions.

### Strengths

To our knowledge, our study is the first ED-focused needs assessment for the LGBTQ+ community. We received a significant number of responses from both sexual and gender minorities with notable socioeconomic diversity.

## LIMITATIONS

While our sample size was robust, it lacked a sufficient number of racially diverse participants to examine the role of race/ethnicity in ED avoidance or discrimination. A limited number of heterosexual cisgender respondents limited comparison to the general population. Limited numbers of respondents reporting seeking care for sexual health (n = 6) or mental health (n = 17) concerns preclude analysis of outcome quality for patients presenting with these complaints. Our sample’s composition of members of a Colorado-based LGBTQ+ organization’s social network reduces applicability to states with differing political climates and LGBTQ+ acceptance pervasiveness.

Additionally, all outcomes were self-reported and retrospective. This raises the possibility of recall bias, where those reporting negative ED encounters remembered greater detail about experiences than those reporting less remarkable visits. Mean time since last visit was five years, an interval that likely reduced recall of specific factors contributing to visit quality and likelihood of identity disclosure. Finally, as our sample was taken from membership within a LGBTQ+ organization, it is very possible that responder bias may have skewed both the response from under-represented groups and the cisgender heterosexual population in our sample, and may not reflect the experiences of the cisgender heterosexual population in general. Similarly, by using our population of sexual minority males as our control group for a subset of our analysis, we may have skewed our results; however, this variation from standard is another reminder that “heterosexual” should not be the default in all circumstances. Doing so allowed us to stratify rates of avoidance among sexual and gender minorities when a convenience sample of cisgender heterosexual respondents was limited.

### Future Studies

Our study revealed significant findings concerning the LGBTQ+ community that may form the basis for future investigation. Future studies administering patient surveys immediately after ED visits may reduce the effect of recall bias and yield more robust data. Investigations seeking to further characterize perceived shortcomings of ED visits in specific areas (eg, provider communication, partner involvement, physical exam, etc) may identify additional deficits in care. Incorporating physician and nurse gender into the analysis might yield informative insight on the effect of provider gender on care. Outcomes data for ED visits including bounce-back rates, hospital admission rates, and mortality might provide further detail on the impact of ED avoidance and intra-ED discrimination. Finally, given the disproportionate burden of ED avoidance and discrimination, future work should further characterize specific deficits impacting the gender minority community. Based on our findings, interventions targeting this population’s care would likely have a powerful impact.

## CONCLUSION

Gender minorities are more likely than sexual minorities to report ED avoidance and discrimination at last ED visit. Future work should further characterize deficits in ED care for this population and assess the efficacy of interventions to reduce ED avoidance and perceived discrimination.

## Figures and Tables

**Table 1:** Summary characteristics of the survey sample.

	Heterosexual cisgender male	Heterosexual cisgender female	Sexual minority male	Sexual minority female	Gender minority	Total
N	6	36	168	150	90	450
Age mean (SD)	69.3 (14.8)	50.7 (19.5)	46.4 (14.8)	41.1 (16.2)	41.0 (17.4)	44.3 (16.7)
Time since last visit mean (SD)	11 (19.6)	7.7 (8.0)	6.2 (6.7)	4.7 (6.1)	3.7 (5.0)	5.3 (6.7)
Any racial minority N (%)	0	5 (14%)	25 (15%)	16 (11%)	9 (10%)	55 (12%)
Income						
< $35,000	4 (67%)	13 (37%)	45 (27%)	50 (34%)	50 (56%)	162 (37%)
$35,000 – $74,999	1 (16.5%)	15 (43%)	52 (32%)	63 (42%)	29 (33%)	160 (36%)
> $75,000	1 (16.5%)	7 (20%)	68 (41%)	35 (24%)	10 (11%)	121 (27%)
Insurance type						
Private	2 (33%)	23 (64%)	129 (77%)	109 (73%)	49 (54%)	312 (69%)
Medicaid	0	3 (8%)	12 (7%)	13 (9%)	17 (19%)	45 (10%
Medicare	3 (50%)	8 (22%)	16 (9%)	15 (10%)	8 (9%)	50 (11%)
Military	1 (17%)	0	4 (2%)	2 (1%)	7 (8%)	14 (3%)
Uninsured	0	1 (3%)	4 (2%)	5 (4%)	2 (2%)	13 (3%)
Multiple	0	1 (3%)	1 (1%)	2 (1%)	4 (4%)	8 (2%)
Other	0	0	2 (1%)	2 (1%)	3 (3%)	7 (2%)

*SD*, standard deviation.

**Table 2:** Odds of ever avoiding care generated by logistic regression.

	N (%) Ever avoided care	Odds Ratio	95% CI	P value
Gender/Sexual orientation				
Male sexual minority	17 (10%)	1 (control)		
Female sexual minority	38 (25%)	3.3	(1.7 – 6.4)	0.001
Transgender man^1^	11 (50%)	6.1	(2.1 – 18)	0.001
Transgender woman^2^	14 (41%)	6.7	(2.6 – 17)	<0.001
Other gender minority	19 (56%)	9.2	(3.7 – 23)	<0.001
Any gender minority^3^	44 (49%)	3.8	(2.2 – 6.6)	<0.001
Income				
<$35,000	54 (37%)	1 (control)		
$35,000 – $74,999	29 (20%)	0.36	(0.19 – 0.70)	0.003
> $75,000	14 (14%)	0.29	(0.14 – 0.64)	0.002
Insurance				
Private	65(23%)	1 (control)		
Medicaid	17 (40%)	0.78	(0.33 – 1.9)	0.574
Medicare	4 (10%)	0.23	(0.070 – 0.73)	0.013
Military	5 (38%)	1.1	(0.30 – 4.4)	0.838
Uninsured	2 (17%)	0.24	(0.045 – 1.3)	0.101
Other	1 (14%)	0.21	(0.022 – 2.0)	0.171
Multiple	4 (57%)	2.0	(0.32 – 13)	0.459
Race				
White (control)	83 (23%)	1		
Other race/ethnicity	16 (31%)	1.9	(0.90 – 4.0)	0.090

P_model_ < 0.0001, P_seudo_ R^2^ = 0.1676.

1 Female to male transgender, a gender minority.

2 Male to female transgender, a gender minority.

3 Any gender minority combines transgender + other gender minority; values generated from a separate logistic regression.

*CI*, confidence interval.

**Table 3:** Number of patients reporting having ever avoided the emergency department by sexuality and gender identity.

	Cisgender Heterosexual N (%)*	Sexual Minority N (%)*	Gender Minority N (%)*	P-value
Ever avoided	6 (14%)	55 (17%)	44 (49%)	<0.001
Financial reasons	3 (50%)	27 (49%)	18 (43%)	0.900
Fear of discrimination	0	8 (15%)	19 (43%)	0.002
Prior negative ED experience	2 (33%)	16 (29%)	20 (45%)	0.241
Prior negative experience outside ED	0	8 (15%)	11 (25%)	0.248

* Subgroups presented as a percentage of those who ever avoided care.

*ED*, emergency department.

**Table 4:** Emergency department factors noted present by the percentage of those who disclosed gender/sexual identity.

ED factor	N (%) Identity disclosed	P (level)
Nurse not commented on	67 (34%)	< 0.001
Supportive nurse	35 (61%)	
Negative nurse	14 (53%)	
Physician not commented on	75 (35%)	0.004
Supportive physician	30 (59%)	
Negative physician	11 (55%)	
Bathroom not commented on	97 (40%)	0.435
Gender-neutral bathroom	3 (33%)	
No gender-neutral bathroom	16 (52%)	
Intake form not commented on	75 (37%)	0.090
Inclusive intake form	9 (45%)	
Non-inclusive intake form	32 (52%)	
LGBTQ+ signage not commented on	86 (40%)	0.173
LGBTQ+ signage	0	
No LGBTQ+ signage	30 (46%)	

*ED*, emergency department; *LGBTQ+*, lesbian, gay, bisexual, transgender, queer, other.

**Table 5:** Odds of reporting last emergency department experience as negative by patient and ED factors.

	N (%) Negative last ED visit	OR	95% CI	*P* value
Unadjusted model				
Gender minority	22 (39%)	11	(5.0–24)	< 0.001
*P_model_ < 0.0001, pseudo R^2^ = 0.1809*				
Adjusted model				
Gender minority		13	(4.6 – 37)	< 0.001
Nurse not commented on		1		
Supportive nurse		0.59	(0.11 – 3.2)	0.527
Negative nurse		22	(5.1 – 92)	< 0.001
Physician not commented on		1		
Supportive physician		NA		
Negative physician		4.4	(1.1 – 18)	0.035
*P_model_ < 0.0001, pseudo R^2^ = 0.50*.				

*ED*, emergency department; *OR*, odds ratio; *CI*, confidence interval.
